# Metallo-β-lactamase-producing *Pseudomonas aeruginosa* Isolates from Two Tertiary Care Centres in a District of Nepal: A Descriptive Cross-sectional Study

**DOI:** 10.31729/jnma.8498

**Published:** 2024-03-31

**Authors:** Pushpa Man Shrestha, Hari Prasad Kattel, Sangita Sharma, Pratibha Bista, Bhupendra Kumar Basnet, Prakash Ghimire, Komal Raj Rijal

**Affiliations:** 1 Central Department of Microbiology, Tribhuvan University, Kirtipur, Kathmandu, Nepal; 2 Department of Microbiology, Tribhuvan University Teaching Hospital, Maharajgunj, Kathmandu, Nepal; 3 Department of Microbiology, Maharajgunj Medical Campus, Maharajgunj, Kathmandu, Nepal; 4 Department of Pathology, Bir Hospital, Mahabaudha, Kathmandu, Nepal; 5 National Academy of Medical Sciences, Bir Hospital, Mahabaudha, Kathmandu, Nepal

**Keywords:** *metallo-ß-lactamase*, *multidrug resistance*, *Pseudomonas aeruginosa*

## Abstract

**Introduction:**

*Pseudomonas aeruginosa* isolates producing metallo-β-lactamase have caused nosocomial outbreaks, severe infections, and ineffective carbapenem therapy worldwide since 1991. Due to their prevalence, hospital infection control techniques are difficult. This study aimed to find out the prevalence of metallo-β-lactamase among *P. aeruginosa* isolates from two tertiary care hospitals in Kathmandu.

**Methods:**

A descriptive cross-sectional study was conducted at the Department of Microbiology and Department of Pathology of two tertiary care centres in Kathmandu from 7 December 2021 to 6 April 2023, after receiving ethical approval from the Ethical Review Board. Isolated strains were identified and tested for antibiotic susceptibility by modified Kirby-Bauer Methods. Metallo-β-lactamase presence was confirmed using an imipenem-imipenem/ethylenediaminetetraacetic acid disc. A convenience sampling method was used. The point estimate was calculated at 95% Confidence Interval.

**Results:**

Among 255, *Pseudomanas aeruginosa* isolates, the distribution of metallo-ß-lactamase-producing *Pseudomanas aeruginosa* was 103 (40.39%) (34.32-46.69 at 95% Confidence Interval). Multidrug resistance categories included multidrug resistance 74 (71.80%), extensively drug resistance 32 (31.10%), *P. aeruginosa* difficult-to-treat 16 (15.53%) and carbapenem-resistant *P. aeruginosa* was determined to be 82 (79.60%).

**Conclusions:**

The study found a high prevalence of metallo-ß-lactamase-producing *Pseudomanas aeruginosa* isolates, requiring early identification, infection control measures, and an all-inclusive antimicrobial therapy protocol to reduce their spread in medical settings.

## INTRODUCTION

The first recorded documentation of *Pseudomonas aeruginosa* isolates producing metallo-ß-lactamase (MBL) was reported in Japan in 1991. Following this, these isolates have been documented in several regions worldwide, including Asia, Europe, Australia, South America, and North America.^1^

*Pseudomonas aeruginosa* isolates that produce MBL have been linked to several nosocomial outbreaks in tertiary healthcare facilities worldwide. These isolates have also been implicated in severe infections, including septicaemia and pneumonia, and have been linked to the ineffectiveness of carbapenem therapy. Hospital infection control strategies face a significant challenge from the prevalence of *Pseudomonas aeruginosa* that produces MBL.^2,3^

This study aimed to find out the prevalence of metallo-β-lactamase among *Pseudomonas aeruginosa* isolates from two tertiary care hospitals in Kathmandu.

## METHODS

A descriptive cross-sectional study was conducted among *Pseudomonas aeruginosa* isolates at Bir Hospital, Mahabaudha, Kathmandu, Nepal and Tribhuvan University Teaching Hospital (TUTH), Maharajgunj, Kathmandu, Nepal from 7 December 2021 to 6 April 2023. The isolates were acquired from a hospital following the receiving of ethical approval for the study from the Ethical Review Board, Nepal Health Research Council, Kathmandu, Nepal (Registration number: 78/2021), Institutional Review Board, National Academy of Medical Sciences, Bir Hospital, Mahabaudha, Kathmandu, Nepal (Reference number: 481/2078/79), and Institutional Review Committee, Institute of Medicine, Maharajgunj, Kathmandu, Nepal (Reference number: 95 (6-11) E2 79/80). The convenience sampling technique was used. All *Pseudomonas aeruginosa* isolates were included.

The sample size was calculated using the formula:


$$\begin{array}{ll} \text{n} & ={\text{Z}}^{2}\times \frac{\text{p}\times \text{q}}{{\text{e}}^{\text{2}}}\\ & =1.96^{2}\times \frac{0.2075\times 0.7925}{0.05^{2}}\\ & =253 \end{array}$$

Where,

n = minimum required sample size
Z = 1.96 at 95% Confidence Interval (CI)
p = prevalence of MBL-*Pseudomonas aeruginosa* isolates from similar study, 20.75%^4^
q = 1-p
e = margin of error, 5%

The required minimum sample size calculated was 253. A total of 255 clinical isolates of *Pseudomonas aeruginosa* were consecutively obtained from clinical specimens (e.g., blood, urine, pus, sputum and others) at different wards. Identifying isolated organisms involved obtaining a pure culture from the original culture. This was achieved using a purity plate and subjecting the acquired culture to various biochemical tests, such as Gram's staining, catalase, and oxidase assays. These tests were conducted to identify the isolated colonies.^5,6^

The antibiotic susceptibility test involved the use of the modified Kirby-Bauer disc diffusion method to assess the susceptibility of clinical isolates to antibiotics. This testing was conducted on Mueller-Hinton agar medium following the guidelines provided by the Clinical and Laboratory Standards Institute (CLSI).^7^ A total of seventeen antibiotics with anti-pseudomonal properties, originating from eight distinct types of antibiotic categories, were applied for experimental evaluation.^8^ The antibiotic discs that were tested in this study included amikacin (AK, 30μg), gentamycin (GEN, 10μg), tobramycin (TOB, 10μg), nettilin (NET, 30μg), imipenem (IMP, 10μg), meropenem (MRP, 10μg), doripenem (DOR, 10μg), ceftazidime (CAZ, 30μg), cefepime (CPM, 30μg), ciprofloxacin (CIP, 5μg), levofloxacin (LE, 5μg), piperacillin/tazobactam (PIT, 100μg/10μg), ticarcillin/clavulanic acid (TCC, 75/10μg), aztreonam (AT, 30μg), fosfomycin (FO, 200μg), polymyxin B (PB, 300 units), and colistin (CL, 10μg) [Hi Media Laboratories Pvt. Ltd., Mumbai, India]. The diameters of the zones were measured and subsequently interpreted following the recommendations provided by the Clinical and Laboratory Standards Institute (CLSI).^7,9,10^ The colistin susceptibility was assessed using the colistin broth disc elution (CBDE) technique. In summary, four MacCartney bottles containing 10 ml of CAMHB were designated as follows: growth control (GC), 1 μg/ml, 2 μg/ml, and 4 μg/ml. Subsequently, colistin discs containing 10 pg each (HiMedia Laboratories Pvt. Ltd., India) were introduced into each container, resulting in a final concentration of 0 μg/ml. Following a 1-minute vortexing period, the bottles were incubated at room temperature for 30 minutes to facilitate the elution of colistin from the discs. Fresh colonies of an overnight growth on Mueller Hinton agar (MHA, HiMedia Laboratories Pvt. Ltd.) supplemented with normal saline (corresponding to a 0.5 McFarland standard) were used to produce the inoculum. A gentle vortex was performed after adding 50 μl of inoculum to each bottle. The turbidity of the growth in the tubes was measured after an overnight incubation at 350C. Furthermore, Polymyxin B susceptibility was performed with a similar approach.^7^ The control organism applied for the antibiotic susceptibility test was *Pseudomonas aeruginosa* ATCC 27853.

The first screening test used Ceftazidime (CAZ) and imipenem (10 μg) to produce MBL. A possible source of MBL was identified by the Clinical and Laboratory Standards Institute (CLSI) if the zone of inhibition measured 18 mm for ceftazidime (CAZ) and/or 19 mm for imipenem (IPM).^7^ In the phenotypic confirmatory test for MBL detection, a Mueller Hinton agar plate was used. Two discs, one containing imipenem (10 μg) and the other containing imipenem/EDTA (IE), were positioned 20 mm apart and in the centre of the plate. The plate was then incubated at 37°C for 24 hours. An observed discrepancy of at least 7 mm in zone diameters between the imipenem and IE discs was considered indicative of an isolate that is positive for MBL.^11^

The data was entered into Microsoft Excel 2021 and analysed. The point estimate was computed along with a 95% CI.

## RESULTS

Among 255 *Pseudomonas aeruginosa* isolates, the distribution of MBL-producing *Pseudomonas aeruginosa* was 103 (40.39%) (34.32-46.69, 95% CI). Among the 103 MBL-*Pseudomonas aeruginosa,* 67 (65.04%) aeruginosa isolates originated from male patients, while the remaining 36 (34.95%) were obtained from female patients. The study showed that 44 (42.70%) MBL-*Pseudomonas aeruginosa* isolates were obtained from the OutPatient Department (OPD) and 40 (30.80%) from the wards. Additionally, the examination of the specimen indicated that MBL- *Pseudomonas aeruginosawas* isolated from pus in 46 (44.66%) of the total isolates (Table 1).

**Table 1:** Distribution of *aeruginosa* isolates based on and specimen (n = 103).MBL- *Pseudomonas* gender, age, origin, and specimen (n = 103).

Variables	n (%)
**Gender**	
Male	67 (65.04)
Female	36 (34.95)
**Age**	
Children (<14 years)	6 (5.82)
Adults (15-60 years)	78 (75.72)
Elderly (>60 years)	19 (18.44)
**Origin**	
OPD	44 (42.71)
Wards	40 (38.83)
ICU	19 (18.44)
Specimen	
Pus	46 (44.66)
Sputum	29 (28.15)
Urine	24 (23.30)
Blood	4 (3.88)

The resistance rates for antipseudomonal cephalosporins showed high resistance at 102 (99.02%), while piperacillin and tazobactam showed relatively low resistance at 23 (22.33%) compared to another antimicrobial category (Table 2).

**Table 2:** Antibiotic susceptibility pattern of MBL- Pseudomonas aeruginosa (n = 103).

Antimicrobial agents	Resistance n (%)	Sensitive n (%)
Aminoglycosides	58 (56.31)	45 (43.68)
Gentamicin	48 (46.60)	55 (53.39)
Tobramycin	44 (42.71)	59 (57.28)
Amikacin	44 (42.71)	59 (57.28)
Nettilin	45 (43.68)	58 (56.31)
Antipseudomonal carbapenems	82 (79.61)	21 (20.38)
Imipenem	79 (76.69)	24 (23.30)
Meropenem	62 (60.19)	41(39.80)
Doripenem	48 (46.60)	55 (53.39)
Antipseudomonal cephalosporins	102 (99.02)	1 (0.97)
Ceftazidime	102 (99.02)	1 (0.97)
Cefepime	59 (57.28)	44 (42.71)
Antipseudomonal fluoroquinolones	66 (64.07)	37 (35.92)
Ciprofloxacin	45 (43.68)	58 (56.31)
Levofloxacin	66 (64.07)	37 (35.92)
Antipseudomonal penicillins + ß- lactamase inhibitors	65 (63.10)	38 (36.90)
Ticarcillin-clavulanic acid	62 (60.19)	41 (39.80)
Piperacillin-tazobactam	23 (22.33)	80 (77.66)
Monobactams-Aztreonam	45 (43.68)	58 (56.31)
Phosphonic acids-Fosfomycin	32 (31.06)	71 (68.93)
Polymyxins	-	103 (100)
Colistin	-	103 (100)
PolymyxinB	-	103 (100)

Out of the 103 isolates of MBL-*Pseudomonas aeruginosa,* 74 (71.84%) demonstrated multi-drug resistance (MDR). Additionally, 32 (31.06%) exhibited extensively established drug resistance (XDR). Furthermore, it was noted that 16 (15.53%) isolates displayed the existence of *Pseudomonas aeruginosa* difficult-to-treat resistance (DTR PA).

## DISCUSSION

The study identified the presence of MBL-*Pseudomonas aeruginosain* 103 (40.39%) isolates. Multiple studies have shown a diverse range of detection frequencies for MBL, ranging from 7% to 65%. A distinct investigation conducted in Kathmandu revealed that *Pseudomonas aeruginosa* accounted for around 16.40% to 20.75% of MBL producer.^4,12^

The present investigation examined the antimicrobial resistance pattern of MBL-Pseudomonas *aeruginosa* isolates. Notably, the highest resistance level was observed against ceftazidime 102 (99.02%), followed by levofloxacin 66 (64.07%). These findings align with previous reports from Nepal, which documented resistance rates exceeding 60%.^13^ As compared to rates reported in Nepal and Saudi Arabia, resistance to cefepime 59 (57.28%), imipenem 79 (76.69%), meropenem 62 (60.19%), gentamycin 48 (46.60%), ciprofloxacin 45 (43.68%), amikacin 44 (42.71%), and piperacillin/tazobactam 23 (22.33%) was found to be higher in this study.^14,15^ Furthermore, it was found that resistance to ticarcillin-clavulanic acid 62 (60.19%) and aztreonam 45 (43.68%) was higher than the rates reported in Nepal, which range from 32.5-46%.^16^ Many studies have estimated varying degrees of colistin and polymyxin B resistance. In the context of our investigation, it was observed that all isolates exhibited full susceptibility to colistin and polymyxin B.^14,17^ In this study, the rate of resistance to fosfomycin was 32 (31.06%), which is lower (i.e., 33.3%) than a study from France.^18^ The results of this study suggest that despite the development of extensive resistance to anti-pseudomonal antibiotics, MBL- *Pseudomonas aeruginosa* may still be susceptible to the following: piperacillin/tazobactam, fosfomycin, amikacin, doripenem, tobramycin, gentamicin, nettilin, ciprofloxacin, aztreonam, colistin and polymyxin B.

MDR rates in Nepal have been calculated by numerous studies and range from 21 to 89%.^14,16,19^ In the current study among the MBL-Pseudomonas *aeruginosa,* 74 (71.84%) MDR isolates were found. A Nepal study found a similar incidence of MDR in *Pseudomonas aeruginosa*, but it's challenging to pinpoint trends due to varying percentages of resistance levels. The study excluded fully susceptible antibiotics, raising the possibility of widespread resistance. The majority of drug-resistant isolates came from inpatients and invasive sites, worsening infection control failure and iatrogenic transmission.^16^

It is quite concerning when carbapenem resistance appears and spreads because it reduces the variety of available treatments. In the current study, 82(79.61%) isolates were non-susceptible to the carbapenems used among MBL producers. The frequency of carbapenem-resistant *Pseudomonas aeruginosa* in the present study was higher than the rate reported in a prior study.^20^

In the current study, the phenotypic technique was applied to identify MBL. The lack of a molecular method for identifying the responsible gene sequence limited the investigation.

## CONCLUSIONS

The study found a high prevalence of MBL-producing *Pseudomonas aeruginosa* isolates, requiring early identification, infection control measures, and an allinclusive antimicrobial therapy protocol to reduce their spread in medical settings. MBL detection remains contentious, but clinical laboratories need a quick method to identify resistant *Pseudomonas aeruginosa.*
